# Intraoperative quantitative assessment of parathyroid blood flow during total thyroidectomy using indocyanine green fluorescence imaging ‐ surgical strategies for preserving the function of parathyroid glands

**DOI:** 10.1002/lio2.868

**Published:** 2022-07-18

**Authors:** Keisuke Iritani, Masanori Teshima, Hikari Shimoda, Hirotaka Shinomiya, Naoki Otsuki, Ken‐ichi Nibu

**Affiliations:** ^1^ Department of Otolaryngology‐Head and Neck Surgery Kobe University Graduate School of Medicine Kobe Japan

**Keywords:** autofluorescence, indocyanine green, near‐infrared, parathyroid gland, thyroidectomy

## Abstract

**Objective:**

We investigated the factors affecting postoperative parathyroid gland (PTG) function and devised an objective index to predict the postoperative PTG function during total thyroidectomy.

**Method:**

This was a retrospective clinical review of 21 consecutive patients who were diagnosed with papillary thyroid carcinoma and underwent total thyroidectomy. The maximum intensity ratio (MIR) was determined as the maximum fluorescence intensity after ICG injection divided by the intensity before ICG injection.

**Results:**

Postoperative hypoparathyroidism is significantly associated with simultaneous central neck dissection (CND) and lateral neck dissection (LND) (*p* = .032). The Spearman correlation test showed a moderate correlation between the MIR and iPTH levels (*p* = .0047). The optimal MIR cutoff value for predicting postoperative hypoparathyroidism was 2.14 with area under the curve = 0.904 (sensitivity: 0.769 and specificity: 1.00).

**Conclusion:**

CND + LND was significantly associated with postoperative hypoparathyroidism. MIR was found useful in predicting the postoperative PTG function.

**Level of Evidence: 3b.:**

## Graphical abstract

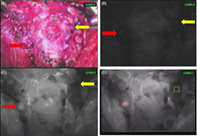

## 1 INTRODUCTION

Hypoparathyroidism is a common postoperative complication of thyroid surgery.^1^, ^2^ Maintaining the blood flow to the parathyroid glands (PTGs) during surgery is extremely important for the preservation of functional PTGs.^3^ However, identifying and preserving the blood flow of PTG is often difficult,^4^, ^5^, ^6^ as PTG is very small in size and buried in the paratracheal fatty tissue. Additionally, its color is similar to that of the fatty tissue. Currently, PTG is intraoperatively identified by the surgeon's unaided eyes, and its blood flow is judged by its color during thyroid surgery. Intraoperative frozen section examination is the definitive method for histological confirmation of parathyroid tissue, and intramuscular transplantation after confirmation by this method can be useful in preventing permanent hypoparathyroidism.^7^ However, this procedure can cause vascular damage as a part of the PTG is removed, and its blood flow and function may decline temporarily. Usually, parathyroid function takes several weeks to recover once it declines.^8^

To date, various techniques have been used to identify PTGs during surgery. In 1971, the usefulness of methylene blue for the rapid identification of PTGs was reported.^9^ However, the surgical outcome did not show improvement over visual identification by the surgeons. Furthermore, intravenous administration of methylene blue occasionally causes side effects such as neurotoxicity.^10^ In 2006, a report was made on the use of aminolevulinic acid, a photosensitizer that accumulates in PTG. However, the results were inconsistent, and it has not been put to practical use as it requires isolation from light for 48 h.^11^ In 2008, a radio‐guided method using 99 m technetium‐methoxy isobutyl isonitrile was proposed to identify PTG. However, this method also failed to become popular due to severe radiation exposure.^12^

In 2011, Paras et al. found endogenous autofluorescence in PTG.^13^ Several studies have recently reported the efficacy of detecting endogenous autofluorescence emitted by the PTG using infrared observation devices.^14^, ^15^ PTGs emit endogenous autofluorescence with a peak at 822 nm when illuminated with excitation light at 785 nm.^14^ Similarly, indocyanine green (ICG) has been reported to emit fluorescence with a peak at approximately 830 nm under irradiation with near‐infrared light (760 nm).^16^ Tissues dyed with ICG can be visualized during surgery using a near‐infrared observation device. Currently, this method is widely applied clinically to evaluate arterial blood flow.^17^ While a large number of studies on the use of ICG in PTG preservation have been reported, the objective evaluation method has not yet been established. There are several reports on the usefulness of visual scores to evaluate the perfusion of the PTGs,^18^, ^19^, ^20^, ^21^ but the criteria for the score are based on the subjective judgment of the surgeons.

To develop a safe and reliable procedure to preserve PTG function during total thyroidectomy, we investigated the factors affecting postoperative PTG function and the effectiveness of ICG fluorescence imaging. We describe our detailed surgical procedure, the correlation between postoperative parathyroid function and clinical factors, and the findings of ICG fluorescence imaging in this report. We also propose an objective index to predict the postoperative function of PTG.

## 2 PATIENTS AND METHODS

### 2.1 Patients

This was a retrospective clinical review of 21 consecutive patients who were diagnosed with papillary thyroid carcinoma and underwent total thyroidectomy with bilateral central neck dissection (CND) and in situ preservation of PTG using ICG fluorescence imaging at the Department of Otolaryngology‐Head and Neck Surgery of the Kobe University Hospital from January 2020 to December 2020. In addition to total thyroidectomy with CND, lateral neck dissection (LND) was also performed when clinically indicated. The clinical stages of thyroid cancer were determined according to the eighth edition of the TNM staging system proposed by the UICC International Union Against Cancer.^22^ Patients with an allergy or intolerance to ICG or those who had previous thyroid or parathyroid surgery were excluded.

### 2.2 Surgical procedures for the preservation of parathyroid perfusion

All surgical procedures were performed by experienced thyroid surgeons in our department. Bilateral CND was also performed in all thyroidectomies. The area of dissection was defined as the area bounded by the trachea, common carotid artery, laryngeal inlet of the recurrent nerve, and supraclavicular border. Blood supply to the upper PTG was preserved on the side where no obvious tumor invasion was observed. Once the superior pole of the thyroid was exposed, the localization of the parathyroid glands was confirmed by observing autofluorescence using the near‐infrared camera. Autofluorescence was observed using VISERA ELITE II® (CLV‐S200‐IR, Olympus Corp., Tokyo, Japan) in seven patients and PDE‐neo® (Hamamatsu Photonics, Hamamatsu, Japan) in the other 14 patients. As described by Zhu et al.,^23^ meticulous capsule dissection was performed to preserve the blood supply to the PTGs. In meticulous capsule dissection, the third‐tier blood vessels of the thyroid gland should be treated close to the natural thyroid capsule during its resection. In this procedure, the anterior branch of the superior thyroid artery was ligated, and the main trunk of the superior thyroid artery was preserved. The distal end of the posterior branch of the superior thyroid artery entering the thyroid gland was ligated just adjacent to the thyroid gland. The inferior thyroid artery was ligated at the level of the distal branches, as close to the thyroid gland as possible. If the inferior thyroid artery was close to the tumor or metastatic lymph node, the inferior thyroid artery was ligated at the main trunk. While dissecting the central lymph nodes, branches to the upper PTG from the inferior thyroid artery were exposed and preserved as far as possible. Except in the case of one patient, lower PTGs were dissected together with CND. Dissected parathyroid tissues were auto‐transplanted into the sternocleidomastoid muscle after pathological confirmation using an intraoperative frozen section, if available. Endogenous fluorescence was used as a guide to search for parathyroid tissue in the excised tissue.

### 2.3 ICG fluorescence imaging

**FIGURE 1:**
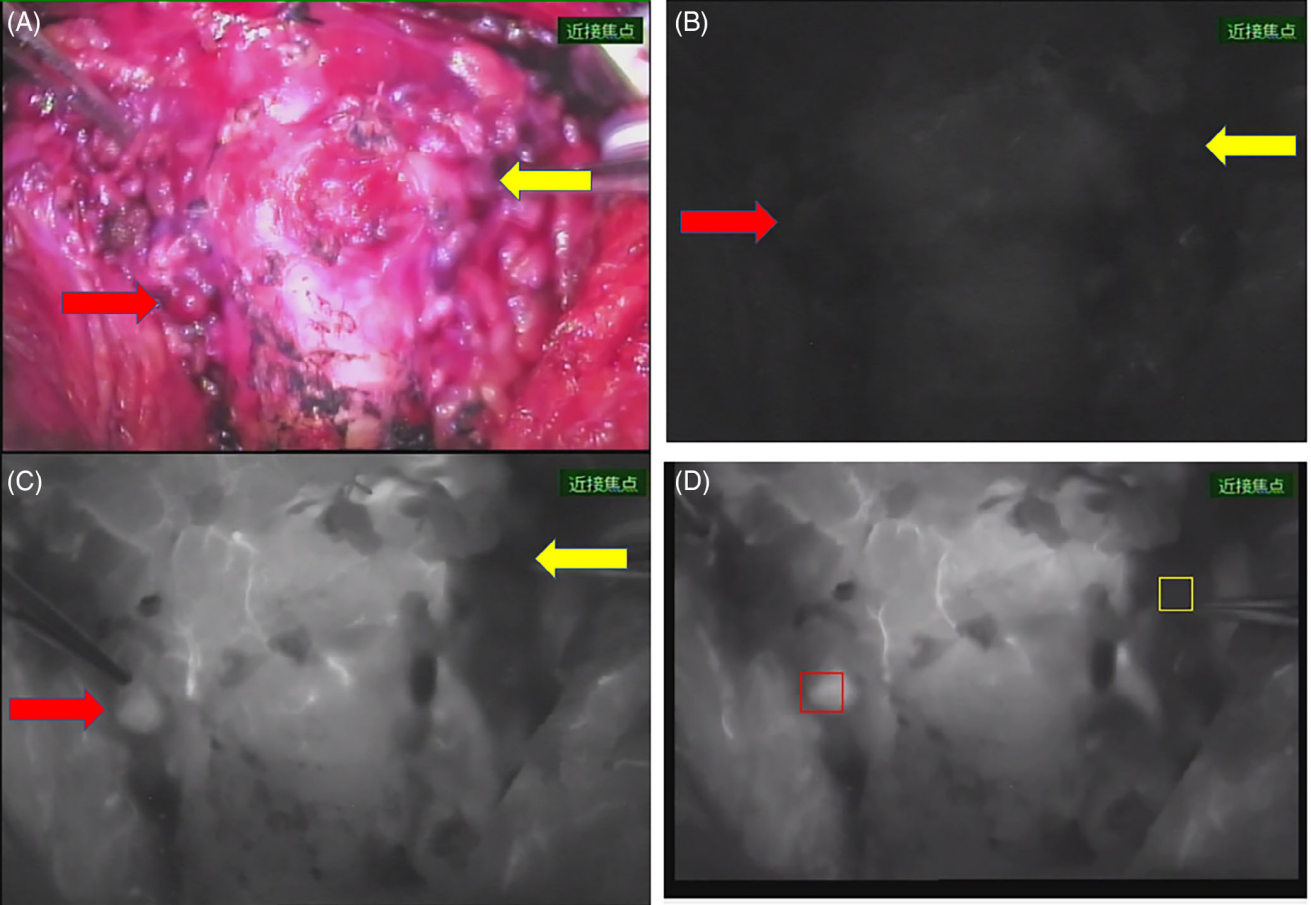
A PDE‐neo image and analysis of fluorescence. Intraoperative photograph of the preserved parathyroid glands with PDE system under white light (A), under infrared observation before ICG injection (B), and after ICG injection (C). Red arrows show the preserved right upper parathyroid gland, and yellow arrows show the left upper parathyroid gland. A screenshot of the fluorescence intensity analysis is shown in (D). Screenshot of the analysis in ROIs. The right upper gland, surrounded by a red frame, shows an increase in luminance after ICG injection. In contrast, the left upper gland surrounded by a yellow frame showed a poor increase in brightness, even after ICG injection.

After the entire thyroid gland was removed, the blood flow to the preserved PTGs was assessed. Initially, preserved PTGs were observed with a near‐infrared camera without ICG. Subsequently, diluted 2.5 mg of ICG (Diagno Green®, Daiichi‐Sankyo, co) was administered intravenously by the anesthesiologist, and the fluorescence intensity of the PTGs was recorded. Autofluorescence and ICG fluorescence were observed using VISERA ELITE II® (CLV‐S200‐IR, Olympus Corp., Tokyo, Japan) in seven patients and PDE‐neo® (Hamamatsu Photonics, Hamamatsu, Japan) in the other 14 patients. The former system emits excitation light at 700–780 nm and acquires fluorescence at wavelengths longer than approximately 820 nm. The latter one emits excitation light at a wavelength of 760 nm and filters out light with a wavelength of less than 820 nm. ROIs® (Hamamatsu Photonics, Hamamatsu, Japan) were used to analyze the intensity of the recorded fluorescence. This software is capable of analyzing the fluorescence intensity of the images and outputting it in real‐time. Intraoperative videos can be analyzed in real‐time, or recorded videos can be analyzed later. The highest fluorescence intensity was observed in the region of the preserved PTGs. The maximum intensity ratio (MIR) was determined as the maximum intensity after ICG injection divided by the intensity before ICG injection. MIR was defined as the highest when multiple PTGs were preserved in situ (Figure 1). In this study, the fluorescence intensity of the PTGs was analyzed using ROIs® and MIR was calculated retrospectively based on the movies obtained during the operations.

### 2.4 Management and follow‐up of the patients

Postoperative serum calcium and intact parathyroid hormone (iPTH) levels were measured by ECLusys “PTH” (Roche Diagnosis K.K, normal range: 15.0–65.0 pg/ml) on the first postoperative day (POD1). Calcium supplementation was administered to all patients on the day of surgery, and adjustments were made according to the levels of serum calcium and iPTH on the following days. Postoperative hypoparathyroidism was defined as an iPTH concentration less than 15 pg/ml on POD1. If hypoparathyroidism was observed on POD1, iPTH was measured again 3 months after the surgery.

### 2.5 Statistical analyses and informed consent

The Spearman correlation test was performed to determine the correlation between two continuous variables with a non‐normal distribution. For comparison of categorical variables, Fisher's exact test was used. To evaluate the predictive ability of the MIR for predicting postoperative PG function, we performed discrimination and calibration analyses. Receiver‐operating characteristic (ROC) curves and area under the curve (AUC) were calculated using a binary logistic model. All statistical analyses were performed using the EZR version 3.5.2. Statistical significance set at *p* < .05 was considered as significant.^24^ All procedures in this study were approved by the Institutional Ethical Committee of Kobe University Hospital (#B210215). Written informed consent was obtained from all the participants.

## 3 RESULTS

### 3.1 Correlation between clinical characteristics and postoperative parathyroid function

Table 1 presents the baseline characteristics of the patients. ICG fluorescence imaging was performed in all patients. The median follow‐up duration was 17 months, with a range of 11–22 months. ICG‐related adverse reactions were not observed. Postoperative hypoparathyroidism was observed in 10 out of 21 patients. Correlations between the baseline characteristics of the patients and postoperative parathyroid function are also summarized in Table 1. Univariate statistical analysis showed that LND (no LND vs. unilateral/bilateral LND) was significantly associated with postoperative parathyroid function.

**TABLE 1:** Baseline characteristics of the study population by postoperative parathyroid hormone status

Factors	Postoperative hypoparathyroidism		*p* values
	+ (*n* = 13)	− (*n* = 8)	
Age			
<55	5	3	>.99
≧55	8	5	
Gender			
Male	8	4	.673
Female	5	4	
Body mass index (kg/m^2^)			
<30	12	6	.531
≧30	1	2	
pT			
T0‐2	5	4	.827
T3	7	3	
T4	1	1	
Lateral neck dissection			
None	3	6	**.032**
unilateral/bilateral	10	2	
In situ preservation of PGs			
Two PGs	4	3	>.99
One PG	9	5	
Auto‐transplanted PGs			
Two PGs	6	2	.535
One PG	3	4	
No PG	4	2	
Device for near‐infrared fluorescence imaging			
PDE‐neo	9	5	>.99
VISERA‐ELITE II	4	3	

Abbreviations: +, intact PTH concentration less than 15 pg/ml on POD1; −, intact PTH concentration 15 pg/ml or more on POD1.

### 3.2 Fluorescence intensity and postoperative parathyroid function

**FIGURE 2:**
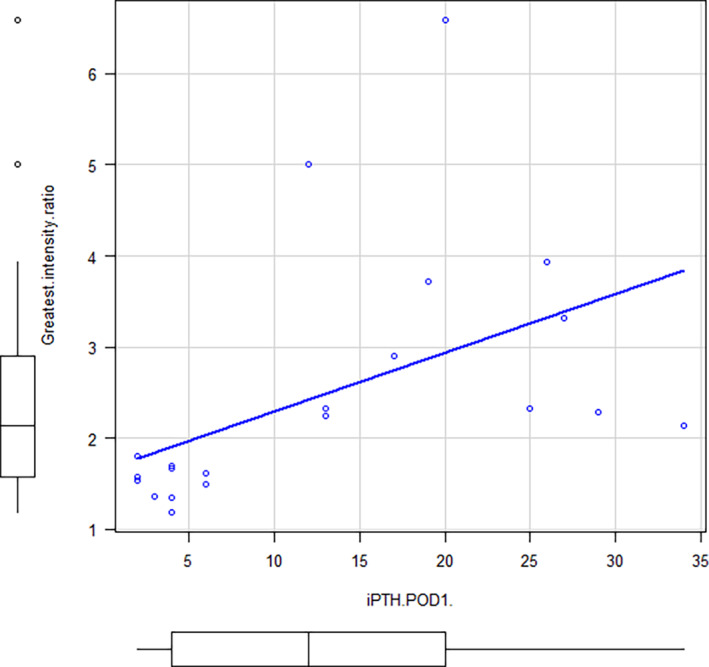
Correlation between Maximum Intensity Ratio and intact PTH. MIR, maximum intensity ratio; iPTH, intact PTH; POD1, the first postoperative day

**FIGURE 3:**
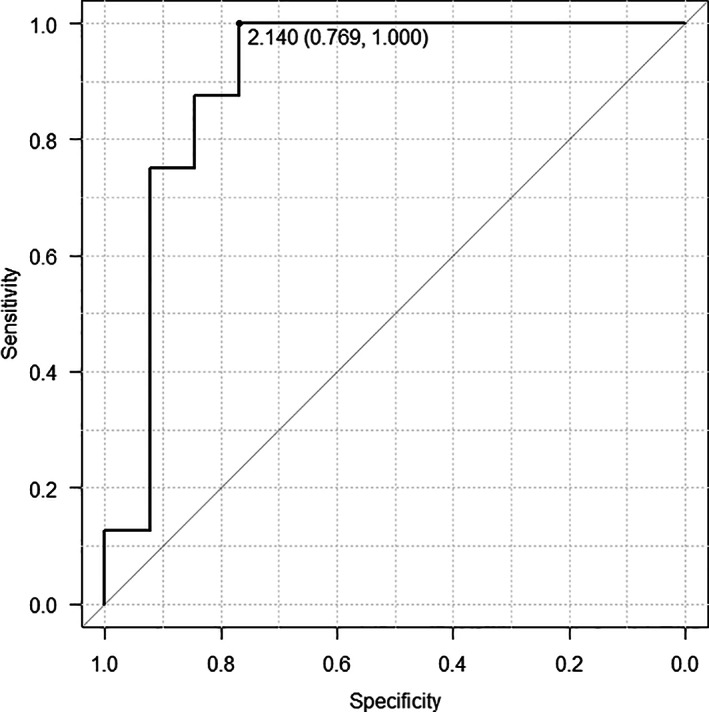
ROC of MIR cutoff value to predict postoperative parathyroid function. The ROC curve analysis demonstrates that the optimal MIR cutoff value is 2.14. The sensitivity was 0.769 and the specificity was 1.00.

Table 2 summarizes the individual clinical/pathological factors, surgical procedures, and findings of fluorescence observation. The Spearman correlation test showed a moderate correlation between the MIR and iPTH levels (Figure 2, rho = 0.455, *p* = .0047). To evaluate the usefulness of MIR for predicting postoperative parathyroid function, we conducted a ROC curve analysis (Figure 3). The optimal MIR cutoff value was 2.14 with AUC = 0.904 (sensitivity: 0.769 and specificity: 1.00).

**TABLE 2:** Individual clinical/pathological outcomes and surgical findings (*n* = 21)

No.	age	gender	BMI	pT	pN	LND	No. of auto‐transplanted PG(s)	No. of excised PG(s)	in‐situ preserved PG(s)	Intensity ratio	Maximum intensity ratio	iPTH (POD1)	iPTH (3 M)	device
1	82	M	24	1b	0	—	1	0	LS	LS: 6.59	6.59	20	—	VISERA ELITEII
2	51	M	32	3	1a	—	0	1	RS, RI	RS: 1.20 RI: 2.14	2.14	34	—	PDE‐neo
3	55	M	32	3	1b	bil	1	1	RS	RS: 1.34	1.34	4	17	VISERA ELITEII
4	81	M	19	3	1a	—	1	0	LS	LS: 2.29	2.29	29	40	VISERA ELITEII
5	71	M	25	3b	1b	R	0	0	LS	LS: 1.49	1.49	6	4	VISERA ELITEII
6	39	M	29	2	1b	R	0	0	LS	LS: 3.31	3.31	27	—	VISERA ELITEII
7	53	M	25	3	1b	R	2	0	LS	LS: 1.19	1.19	4	13	VISERA ELITEII
8	69	F	29	3a	1b	bil	0	3	LS	LS: 1.36	1.36	3	3	VISERA ELITEII
9	55	F	30	1b	1b	L	2	0	RS, LS	RS: 1.34, LS: 1.67	1.67	4	24	PDE‐neo
10	71	F	21	2	1b	bil	2	1	LS	LS: 1.538	1.538	2	6	PDE‐neo
11	77	F	26	3b	1a	—	2	1	LS	LS: 3.72	3.72	19	—	PDE‐neo
12	64	F	23	4a	0	—	2	1	LS	LS: 2.90	2.90	17	—	PDE‐neo
13	75	M	15	3	0	—	2	1	LS	LS: 5	5.00	12	19	PDE‐neo
14	54	M	25	2	1a	—	1	0	RS, LS	RS: 2.32, LS: 1.90	2.32	13	31	PDE‐neo
15	58	F	34	1b	0	—	1	0	RS, LS	RS: 3.93, LS: 1.90	3.93	26	—	PDE‐neo
16	61	M	28	2	1a	L	2	1	RS	RS: 1.61	1.61	6	19	PDE‐neo
17	61	F	29	3	1b	L	1	2	RS, LS	RS: 2.24, LS: 2.17	2.24	13	49	PDE‐neo
18	44	F	23	4a	1b	R	0	1	LS	LS: 1.57	1.57	2	20	PDE‐neo
19	29	F	25	1	1b	R	1	2	RS, LS	RS: 2.33 LS: 1.71	2.33	25	—	PDE‐neo
20	53	M	27	3b	1a	R	0	0	LS	LS: 1.80	1.80	2	5	PDE‐neo
21	42	M	30	2	1b	—	2	0	RS, LS	RS: 1.69, LS: 1.36	1.69	4	22	PDE‐neo

Abbreviations: BMI, body mass index; F, female; iPTH (3 M), intact PTH 3 months after the surgery; iPTH (POD1), intact PTH on the first postoperative day; LND, lateral neck dissection; LS, left superior gland; M, male; PGs, parathyroid glands; RI, right inferior gland; RS, right superior gland.

## 4 DISCUSSION

Hypoparathyroidism is one of the most common postoperative complications in thyroid surgery,^25^ and the risk of hypoparathyroidism is further increased when CND is performed.^26^ Maintaining the blood flow to the PTG during surgery is extremely important for its functional preservation.^3^ The originality of our study lies in the fact that we analyzed the risk factors in hypoparathyroidism on the following day of surgery and proposed an objective index to determine whether superior parathyroid blood flow could be preserved intraoperatively.

Considering the high frequency of central neck lymph node metastasis in papillary thyroid cancer, we perform CND during total thyroidectomy in our hospital. On the other hand, in follicular carcinoma, we do not always perform CND because central lymph node metastasis is extremely rare compared to papillary thyroid carcinoma. Therefore, in the present study, we focused our analysis on patients who underwent total thyroidectomy and CND for papillary thyroid cancer. We investigated the risk factors for postoperative hypoparathyroidism on POD1 in 21 consecutive patients who underwent total thyroidectomy with CND and assessed the efficacy of intraoperative ICG fluorescence imaging to evaluate the blood supply of preserved parathyroid tissue. We show that LND was a risk factor for postoperative hypoparathyroidism. In patients who underwent simultaneous LND with CND, preservation of blood flow during neck dissection may be difficult, consistent with the previous reports.^27^, ^28^

In recent years, a variety of new techniques have been reported to reliably identify PTGs and efficiently preserve their blood flow. Among them, promising results have been reported for autofluorescence specific to parathyroid tissue and ICG fluorescence techniques. PTGs emit endogenous autofluorescence with a peak at 822 nm when illuminated with excitation light at 785 nm, and ICG emits fluorescence at 830 nm when illuminated with excitation light at 760 nm, respectively.^14^ Although the endogenous autofluorescence of the PTGs and the emission light of ICG are different, their wavelengths are close enough that both can be observed with the same infrared device. The two near‐infrared devices used in this study (VISERA ELITE II® and PDE‐neo®) were both developed for the ICG fluorescence method, and both were considered to be able to detect autofluorescence in the parathyroid glands due to their characteristics. In this study, we used the infrared device for the following three purposes: (1) localization of parathyroid glands before resection, (2) search for parathyroid glands for auto‐transplantation from dissected specimens, and (3) evaluation of the blood flow of the in‐situ preserved parathyroid glands.

However, as shown in Table 2, the numbers of detected parathyroid glands in the present series were not as high as we initially expected. Since the present series consisted of advanced thyroid carcinoma, principally, we performed bilateral central part dissection in all patients. As McWade et al.^29^ reported, well‐differentiated thyroid carcinoma reduces parathyroid autofluorescence intensity, making it difficult to distinguish parathyroid glands from lymph nodes or fatty tissue. Thus, some of the inferior parathyroid glands might be resected along with central part tissues without realizing it. However, detection of endogenous autofluorescence emitted by the PTG using infrared observation devices was useful to identify the superior parathyroid gland as described below.

As for the purpose 3, Yavuz et al. proposed a visual score to evaluate the perfusion of the PTGs,^18^ but this score is based on the subjective judgment of the surgeon. Vidal Fortuny et al. reported the usefulness of a randomized clinical trial in patients with adequate parathyroid blood flow as determined by ICG fluorescence scoring.^19^ Thus, the usefulness of ICG fluorescence as a method of assessing parathyroid blood flow has been increasingly reported in recent years. However, an objective index to quantify blood flow was not proposed in these reports.^19^ Noltes et al. proposed an objective measure of ICG fluorescence in the evaluation of parathyroid blood flow.^30^ However, in their study, this index was measured postoperatively, not in real‐time intraoperatively. To establish an objective index to predict intraoperatively the blood flow of PTG, in this study, we focused on the ratio of the maximum luminance before and after ICG infusion using ROIs® intraoperatively. In this study, we showed that MIR, a measure of blood flow in the most perfused parathyroid gland, correlated with PTH levels on postoperative day 1, as shown in Figure 2. There was no correlation between the number of parathyroid glands preserved and the presence of postoperative hypoparathyroidism, as shown in Table 2.

In this retrospective study, two instruments were used due to schedule constraints. Both instruments were developed for ICG fluorescence imaging and have the same feature of acquiring fluorescence above 820 nm. The present results suggest that the ratio of maximum luminance is highly correlated with postoperative parathyroid function. In addition, similar results were obtained with both types of equipment. Taking the ratio of the maximum intensity before and after ICG infusion may be a universally applicable method for evaluating the parathyroid blood flow. The greatest advantage of this method is that it can promptly quantify the blood flow of the preserved PTGs intraoperatively and predict the postoperative parathyroid function with high accuracy. The image analysis software used in this study, ROI®, is a less expensive software manufactured by Hamamatsu Photonics. This software is universally available and can analyze videos and images produced by others other than PDE‐neo®. This software can record ICG fluorescence and measure luminance on the spot and output and graph the results of luminance measurement both intraoperatively and retrospectively based on the obtained videos. Therefore, this analysis can be directly applied intraoperatively, although the MIR calculation was done retrospectively in this study.

We perform bilateral CND simultaneously in total thyroidectomy for papillary thyroid carcinoma. While this has the advantage of controlling central lymph node metastasis, it has the disadvantage that the inferior parathyroid gland cannot be preserved. Therefore, the inferior parathyroid gland is implanted intramuscularly, while this method has prevented permanent hypoparathyroidism in only four cases (Table 2). Based on the results of this study, we propose the following surgical strategy for preserving parathyroid function based on infrared observation with ICG contrast. After exposure to the superior pole of the thyroid gland, the location of the parathyroid gland is confirmed by autofluorescence observed with a near‐infrared camera. After dissection, if the result of ICG fluorescence imaging shows a MIR of 2.14 or higher, blood flow to the preserved PTG can be considered preserved; if the MIR is less than 2.14, blood flow to the anatomically preserved parathyroid glands should be considered inadequate. Regardless of the result of MIR, since it is difficult to preserve blood flow to the inferior parathyroid gland if bilateral CND is performed, the inferior and inadvertently resected superior parathyroid glands should be auto‐transplanted to prevent permanent hypoparathyroidism.

However, we acknowledge that there are several limitations to this study. First, this study was a retrospective study with a limited number of cases. A multi‐institutional prospective study consisting of a large number of patients should be considered to draw definitive conclusions on the usefulness of ICG fluorescence imaging and determine the cutoff value of MIR. Second, the possibility of the presence of ectopic parathyroid glands cannot be excluded. Another limitation is that there were several patients in which the total number of parathyroid glands preserved in situ, intramuscularly implanted, or resected and confirmed in the specimen is less than four. The possible explanations for this finding are (1) failure to preserve the inferior parathyroid glands mainly due to a large number of central zone lymph nodes, (2) intrathyroidal parathyroid glands, and (3) ectopic parathyroid glands. Ideally, four parathyroid glands should be identified in all cases, but this may be difficult due to the possibility of multiple central lymph node metastases in this retrospective study.

## 5 CONCLUSIONS

To develop a safe and reliable strategy to preserve PTG function during total thyroidectomy, we investigated the factors affecting the postoperative PTG function and the effectiveness of ICG fluorescence imaging. Univariate statistical analysis showed that LND was significantly associated with the incidence of postoperative hypoparathyroidism after total thyroidectomy. MIR, determined by the ratio of maximum fluorescence intensity after ICG injection divided by fluorescence intensity before ICG injection, was found useful in predicting the postoperative parathyroid function. When the MIR is less than 2.14, the blood supply of the preserved parathyroid gland should be considered insufficient.

## FUNDING INFORMATION

This research received no specific grant from any funding agency in the public, commercial, or not‐for‐profit sectors.

## CONFLICT OF INTEREST

The authors declare no conflict of interest.
